# Bioinformatics analyses of gene expression profile to identify pathogenic mechanisms for COVID-19 infection and cutaneous lupus erythematosus

**DOI:** 10.3389/fimmu.2023.1268912

**Published:** 2023-10-31

**Authors:** Zhenyu Gao, Xinchao Zhai, Guoqing Yan, Yao Tian, Xia Huang, Qingchao Wu, Lin Yuan, Linchong Su

**Affiliations:** ^1^ Hubei Provincial Key Laboratory of Occurrence and Intervention of Rheumatic Disease, Minda Hospital of Hubei Minzu University, Enshi, China; ^2^ Department of Rheumatology and Immunology, Minda Hospital of Hubei Minzu University, Enshi, China

**Keywords:** COVID-19, cutaneous lupus erythematosus, differentially expressed genes, regulatory network, pathogenic mechanisms

## Abstract

**Objective:**

The global mortality rates have surged due to the ongoing coronavirus disease 2019 (COVID-19), leading to a worldwide catastrophe. Increasing incidents of patients suffering from cutaneous lupus erythematosus (CLE) exacerbations after either contracting COVID-19 or getting immunized against it, have been observed in recent research. However, the precise intricacies that prompt this unexpected complication are yet to be fully elucidated. This investigation seeks to probe into the molecular events inciting this adverse outcome.

**Method:**

Gene expression patterns from the Gene Expression Omnibus (GEO) database, specifically GSE171110 and GSE109248, were extracted. We then discovered common differentially expressed genes (DEGs) in both COVID-19 and CLE. This led to the creation of functional annotations, formation of a protein-protein interaction (PPI) network, and identification of key genes. Furthermore, regulatory networks relating to these shared DEGs and significant genes were constructed.

**Result:**

We identified 214 overlapping DEGs in both COVID-19 and CLE datasets. The following functional enrichment analysis of these DEGs highlighted a significant enrichment in pathways related to virus response and infectious disease in both conditions. Next, a PPI network was constructed using bioinformatics tools, resulting in the identification of 5 hub genes. Finally, essential regulatory networks including transcription factor-gene and miRNA-gene interactions were determined.

**Conclusion:**

Our findings demonstrate shared pathogenesis between COVID-19 and CLE, offering potential insights for future mechanistic investigations. And the identification of common pathways and key genes in these conditions may provide novel avenues for research.

## Introduction

Lupus erythematosus (LE) is identified as an autoimmune condition characterized by enduring inflammation and includes a multitude of subtypes such as systemic lupus erythematosus (SLE) and cutaneous lupus erythematosus (CLE). CLE itself can be further divided into distinct types like acute, subacute, chronic, and intermittent (1). LE’s broad clinical presentations can range from UV-triggered skin rash and widespread hair loss to ulcers, red to discoid plaques, and scar formation (2). Based on a study conducted in Sweden, the incidence of CLE is estimated to be around 4.0 per 100,000 (3). Interestingly, approximately 20% of CLE patients evolve into SLE over a span of three to five years (4).

CLE is a skin-focused autoimmune disorder involving the simultaneous activation of both innate and adaptive immune systems (5). Triggered by a combination of genetic factors and, to some extent, immunostimulatory elements like UV light, it results in an autoimmune response against the skin’s surface layer (6). Hallmarks of this reaction encompass cytotoxic lymphocyte and plasmacytoid dendritic cell (pDC) infiltration into the basal epidermal layer and apoptosis of native keratinocytes. Previous studies confirm that type I/III interferons along with associated cytokines, primarily CXCL10, serve as key proinflammatory components in CLE development (7). Moreover, cytotoxic CXCR3+ lymphocytes are lured to the injury site by the matching chemokine CXCL10, mainly expressed in the lower epidermal layers of active skin lesions, hence leading to keratinocyte cell death (8). Genetic elements significantly contribute to CLE progression. Past investigations highlight the essential link between specific genes, such as HLA subtypes, TNF-α, and complement promoter variants, and an increased propensity for CLE (9, 10). In addition, a recent exhaustive genome-wide association study comparing 183 CLE cases with a control group of 1288 healthy individuals found polymorphisms in two genes, casein kinase 2 and RPP21, displaying a significant association with CLE vulnerability. However, despite recognizing several contributors like autoimmune, genetic, molecular, environmental, and drug-related factors in CLE pathogenesis, research concentrating on the association between COVID-19 and CLE is still scarce.

The epidemic sparked by the SARS-CoV-2 virus first came to light in Wuhan, China, during December 2019. This infectious calamity spread rapidly worldwide, bearing profound impact on both international health status and socio-economic scenarios (11). The World Health Organization (WHO) put forth an alarming estimation of around 14.83 million unanticipated deaths globally, a number that is 2.74 times the 5.42 million deaths officially accredited to COVID-19 in the same timeframe (12). Significantly, the incidence of skin manifestations in COVID-19 patients is projected to fluctuate between 1.8% and nearly 25%, with these skin anomalies surfacing on various body regions (13, 14). COVID-19 can be categorized into acute phase and chronic phase (15). Clinical symptoms may vary from fever, cough to skin lesions, accompanied by changes in pro-inflammatory factors such as IFNs, which share similarities with physiological and pathological characteristics of CLE (15, 16). The transformed symptoms bear a resemblance to those of cutaneous SLC. However, despite recognizing several contributors like autoimmune, genetic, molecular, environmental, and drug-related factors in CLE pathogenesis, research concentrating on the association between COVID-19 and CLE is still scarce.

Currently, there have been no research reports on the clinical outcomes of CLE patients co-infected with COVID-19, nor are there statistical data on COVID-19 prevalence among CLE patients. Nevertheless, these gaps in knowledge do not preclude the potential risks that COVID-19 may pose to individuals with CLE. Recently, reports have cited a growing number of cases characterized by cutaneous lupus flare following COVID-19 infection or COVID-19 immunization (17). Based on the currently available information, it has been suggested that the interplay between the S protein of SARS-CoV-2 and cytoplasmic RNA-binding proteins, coupled with augmented interferon responses induced by COVID-19 vaccination, might potentially contribute to the exacerbation of lupus disease symptoms (18). Nonetheless, the precise mechanism underlying this phenomenon remains incompletely understood.

In pursuit of a novel understanding of the potential common mechanisms between cutaneous lupus erythematosus (CLE) and COVID-19, we embarked on a study aimed at exploring their intersecting transcriptional landscapes and uncovering the pivotal genes tied to CLE exacerbated by COVID-19. We harnessed the information within two datasets from the GEO database (GSE171110 and GSE109248), utilizing a blend of bioinformatics and enrichment analyses to pinpoint shared Differentially Expressed Genes (DEGs) and decipher their functional contributions in both maladies. Additionally, leveraging the STRING database and Cytoscape software (version 3.9.1), we engineered a Protein-Protein Interaction (PPI) network and conducted an in-depth investigation of gene modules to reveal central hub genes. The findings from our research hold substantial importance for understanding the intricate biological cogs that drive these two diseases and could potentially set the stage for exciting future research directions.

## Materials and methods

### Data collection

We obtained the transcriptomic datasets for GSE171110 and GSE109248 from the GEO database (Gene Expression Omnibus, https://www.ncbi.nlm.nih.gov/geo/). The GSE171110 dataset, constructed on the GPL16791 platform, encompasses data from 44 individuals with COVID-19 and 10 healthy subjects. Similarly, the GSE109248 dataset, created on the GPL10558 platform, incorporates data from 25 CLE tissue samples and 14 healthy tissue samples.

### Identification of differentially expressed genes and common DEGs among COVID-19 and CLE

In our quest to identify the DEGs separating COVID-19 and HC subjects within the GSE171110 dataset, we adopted the use of limma packages within the R programming language. By harnessing this statistical tool, we could spotlight genes that fulfilled specific threshold standards: a P value, adjusted for false discovery rate, of less than 0.05 and an absolute log2 fold change (log2FC) reaching 1.0 or surpassing it, thus categorizing them as DEGs. In parallel, we conducted an identical analytical procedure for the GSE109248 dataset to unveil DEGs distinguishing CLE and HC samples. To illuminate the intersection of these datasets, we utilized the VennDiagram package in R, which equipped us to pinpoint the shared DEGs between GSE171110 and GSE109248.

### Functional enrichment analysis of common DEGs

In order to functionally categorize and illustrate the common DEGs, we conducted an enrichment analysis using the GO (19) and KEGG (20) databases. To accomplish this, we employed the ‘clusterProfiler’ and ‘org.Hs.eg.db’ packages in R (21). The enrichment analysis encompassed three ontologies: BP, CC, and MF. By leveraging these resources, we aimed to gain insights into the functional roles and pathways associated with the identified DEGs.

### Construction of protein-protein interaction network

To carry out a thorough PPI network analysis of the pinpointed DEGs, we turned to the STRING database. To guarantee the integrity and reliability of the interactions, we imposed a stringent requirement: the interaction score must exceed 0.4. This analytic endeavor was accomplished using the Search Tool for the Retrieval of Interaction Gene/Proteins (STRING), version 11.5, which is publicly accessible via http://string-db.org/ (22). Following this, we employed the Cytoscape software, specifically its version 3.9.1 (www.cytoscape.org/) (23), as our tool of choice to generate a visual depiction of the PPI network for the DEGs that were consistently identified across our analyses. By harnessing the capabilities of these resources, we were equipped with a graphic interpretation of how the DEGs interacted within the network.

### Identification and network analysis of hub genes

Using CytoHubba (24), an add-on of Cytoscape software, we calculated the hub genes. To identify the final hub genes, we employed five algorithms (Degree, MCC, MNC, Closeness, and EPC) and the UpSetR package in R. Additionally, we used GeneMANIA (http://genemania.org), an online tool, to carry out the network analysis of these discovered hub genes.

### Construction of genes-TFs (transcription factors) regulatory network

To analyze the transcriptional regulatory network (TRN), we collect TF–gene interactions from the TRRUST (Transcriptional Regulatory Relationships Unraveled by Sentence-based Text mining, www.grnpedia.org) database. And the TRN was visualized with Cytoscape software.

### Recognition of gene-miRNA regulatory network

In this study, we utilized miRTarbase databases (https://mirtarbase.cuhk.edu.cn), which describe experimentally validated miRNA-target interactions, to analyze miRNA gene regulations. And the regulatory network was visualized with NetworkAnalyst (www.networkanalyst.ca) online tool.

### Diagnostic value of hub genes

The diagnostic performance of the hub genes for COVID-19 and CLE was assessed separately by constructing ROC curves and calculating the area under the ROC curve (AUC) using the “pROC” R packages.

## Results

Analysis of DEGs and Common DEGs Between COVID-19 and CLE

**Figure 1:**
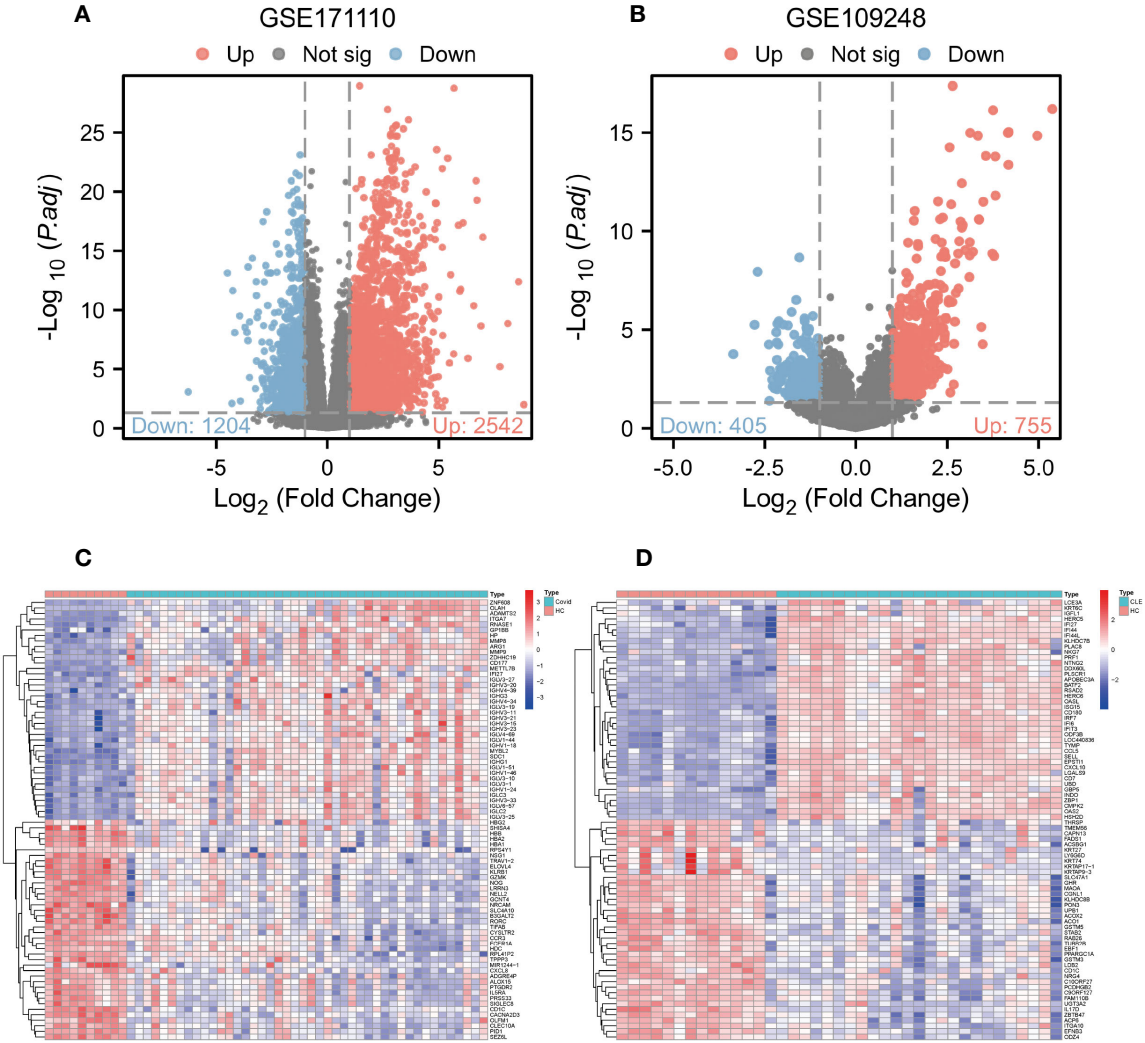
Analysis of gene expression of COVID-19 and CLE **(A)** Volcano plot of DEGs from GSE171110. **(B)** Volcano plot of DEGs from GSE109248. **(C)** The heatmap of GSE171110. **(D)** The heatmap of GSE109248.

**Figure 2:**
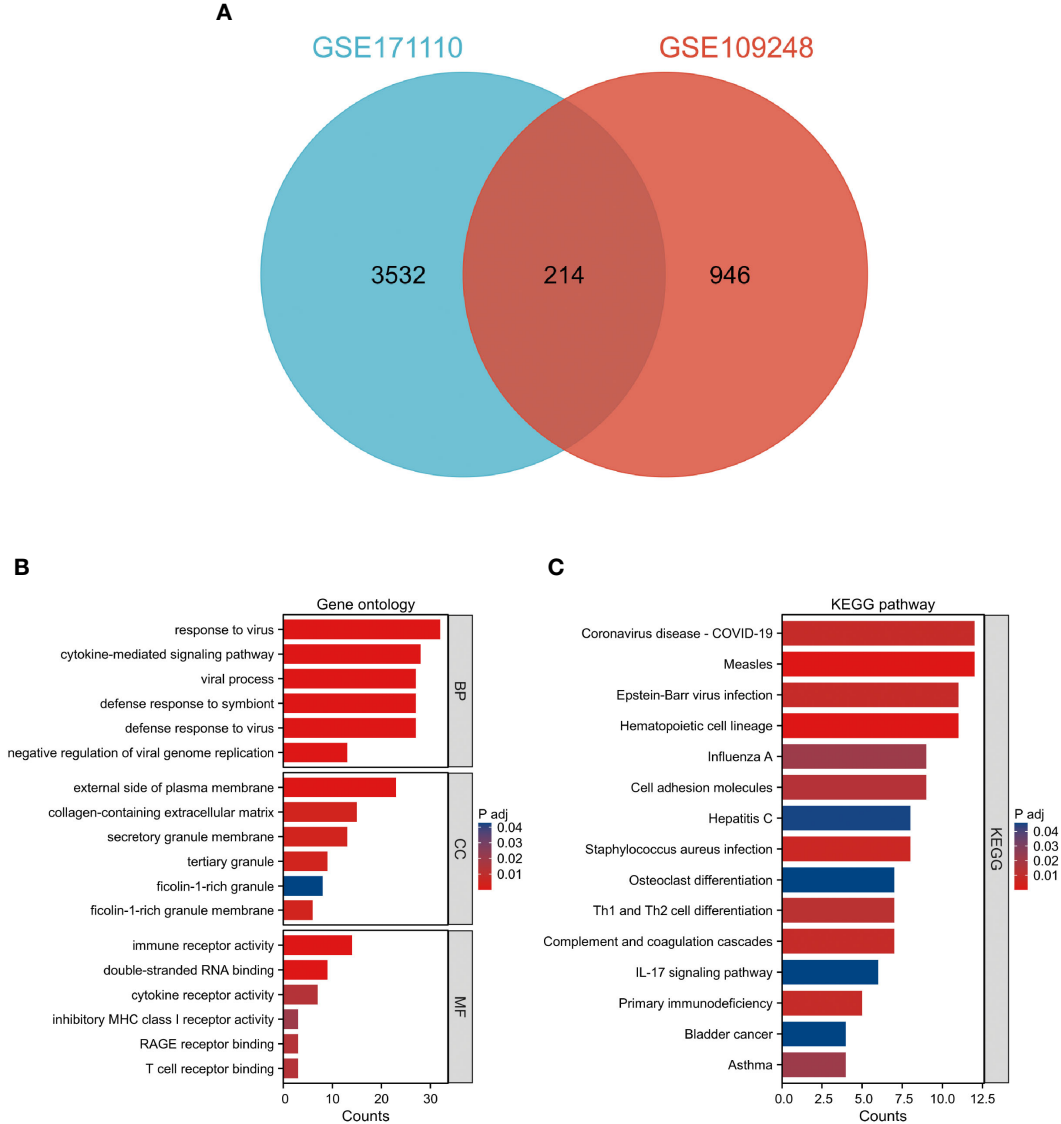
Examination of prevalent DEGs shared between COVID-19 and CLE **(A)** A Venn representation showing overlapping DEGs found in two datasets. **(B)** Evaluation of GO enrichment for the common DEGs. **(C)** Analysis of KEGG pathway enrichment for these mutually identified DEGs. The color distinction signifies the adjusted P-value, while the DEG counts are reflected by the bar’s length.

Comparative analysis of case samples and HC samples allowed us to determine DEGs, using set criteria (adjusted P < 0.05 and |log2FC|≥ 1.0). In the dataset GSE171110, we found 3746 DEGs consisting of 2542 up-regulated and 1204 down-regulated ones (Refer to **Figures 1A, C**). Similarly, in the GSE109248 dataset, we observed 1160 DEGs, with 755 being up-regulated and 405 down-regulated (Refer to **Figures 1B, D**). Subsequently, we pinpointed 214 shared DEGs between GSE171110 and GSE109248 utilizing R’s VennDiagram package (Refer to **Figure 2A**).

### Gene ontology and KEGG pathway enrichment analysis

The GO descriptors were organized into three distinct ontologies: BP, CC, and MF. Through the course of our analysis, the BP ontology revealed a marked enrichment of common DEGs within pathways related to the body’s defensive reaction to viral invasion, signaling mediated by cytokines, and viral processes. Regarding the MF ontology, it was found that there was a significant enrichment of DEGs linked with functions like immune receptor activity, the binding of double-stranded RNA, and cytokine receptor activity. Additionally, in the CC ontology, DEGs were notably linked with locations including the outer layer of the plasma membrane, the collagen-enriched extracellular matrix, and the membrane of secretory granules (Refer to **Figure 2B** and **Table 1**). A group of 15 significant pathways emerged from our KEGG pathway analysis, highlighting a significant presence of common DEGs in conditions such as COVID-19, Measles, infection by the Epstein-Barr virus, Influenza A, and Asthma (See **Figure 2C**) (Refer to **Table 2**).

**Table 1:** Significantly enriched GO terms of common DEGs.

Ontology	ID	Description	GeneRatio	BgRatio	pvalue	p.adjust
BP	GO:0009615	response to virus	32/201	392/18800	2.66917E-19	7.92742E-16
BP	GO:0051607	defense response to virus	27/201	290/18800	7.45774E-18	8.06478E-15
BP	GO:0140546	defense response to symbiont	27/201	291/18800	8.14624E-18	8.06478E-15
BP	GO:0045071	negative regulation of viral genome replication	13/201	57/18800	2.61594E-14	1.94234E-11
BP	GO:0016032	viral process	27/201	418/18800	6.66757E-14	3.96054E-11
MF	GO:0140375	immune receptor activity	14/207	148/18410	1.30215E-09	5.46902E-07
MF	GO:0003725	double-stranded RNA binding	9/207	75/18410	1.59601E-07	3.35161E-05
MF	GO:0004896	cytokine receptor activity	7/207	97/18410	0.000112094	0.01332374
MF	GO:0042608	T cell receptor binding	3/207	10/18410	0.000158616	0.01332374
MF	GO:0050786	RAGE receptor binding	3/207	10/18410	0.000158616	0.01332374
CC	GO:0009897	external side of plasma membrane	23/210	455/19594	7.82588E-10	2.27733E-07
CC	GO:0030667	secretory granule membrane	13/210	312/19594	3.39326E-05	0.004130128
CC	GO:0101003	ficolin-1-rich granule membrane	6/210	61/19594	4.79502E-05	0.004130128
CC	GO:0062023	collagen-containing extracellular matrix	15/210	429/19594	6.39886E-05	0.004130128
CC	GO:0070820	tertiary granule	9/210	164/19594	7.09644E-05	0.004130128

**Table 2:** Significantly enriched KEGG terms of common DEGs.

Ontology	ID	Description	GeneRatio	BgRatio	pvalue	p.adjust
KEGG	hsa04640	Hematopoietic cell lineage	11/124	99/8164	2.58717E-07	5.35544E-05
KEGG	hsa05162	Measles	12/124	139/8164	1.15197E-06	0.000119229
KEGG	hsa05150	Staphylococcus aureus infection	8/124	96/8164	9.79842E-05	0.006760908
KEGG	hsa05171	Coronavirus disease - COVID-19	12/124	232/8164	0.000199581	0.008610924
KEGG	hsa05169	Epstein-Barr virus infection	11/124	202/8164	0.000238253	0.008610924
KEGG	hsa05340	Primary immunodeficiency	5/124	38/8164	0.000250491	0.008610924
KEGG	hsa04610	Complement and coagulation cascades	7/124	85/8164	0.000291191	0.008610924
KEGG	hsa04658	Th1 and Th2 cell differentiation	7/124	92/8164	0.000473494	0.012251658
KEGG	hsa04514	Cell adhesion molecules	9/124	157/8164	0.000615963	0.01416715
KEGG	hsa05164	Influenza A	9/124	171/8164	0.001134379	0.021855094
KEGG	hsa05310	Asthma	4/124	31/8164	0.001161382	0.021855094
KEGG	hsa05160	Hepatitis C	8/124	157/8164	0.002615657	0.045120079
KEGG	hsa04657	IL-17 signaling pathway	6/124	94/8164	0.002980046	0.045897717
KEGG	hsa04380	Osteoclast differentiation	7/124	128/8164	0.003261201	0.045897717
KEGG	hsa05219	Bladder cancer	4/124	41/8164	0.003325922	0.045897717

### Protein-protein interaction network construction and hub gene identification

**Figure 3:**
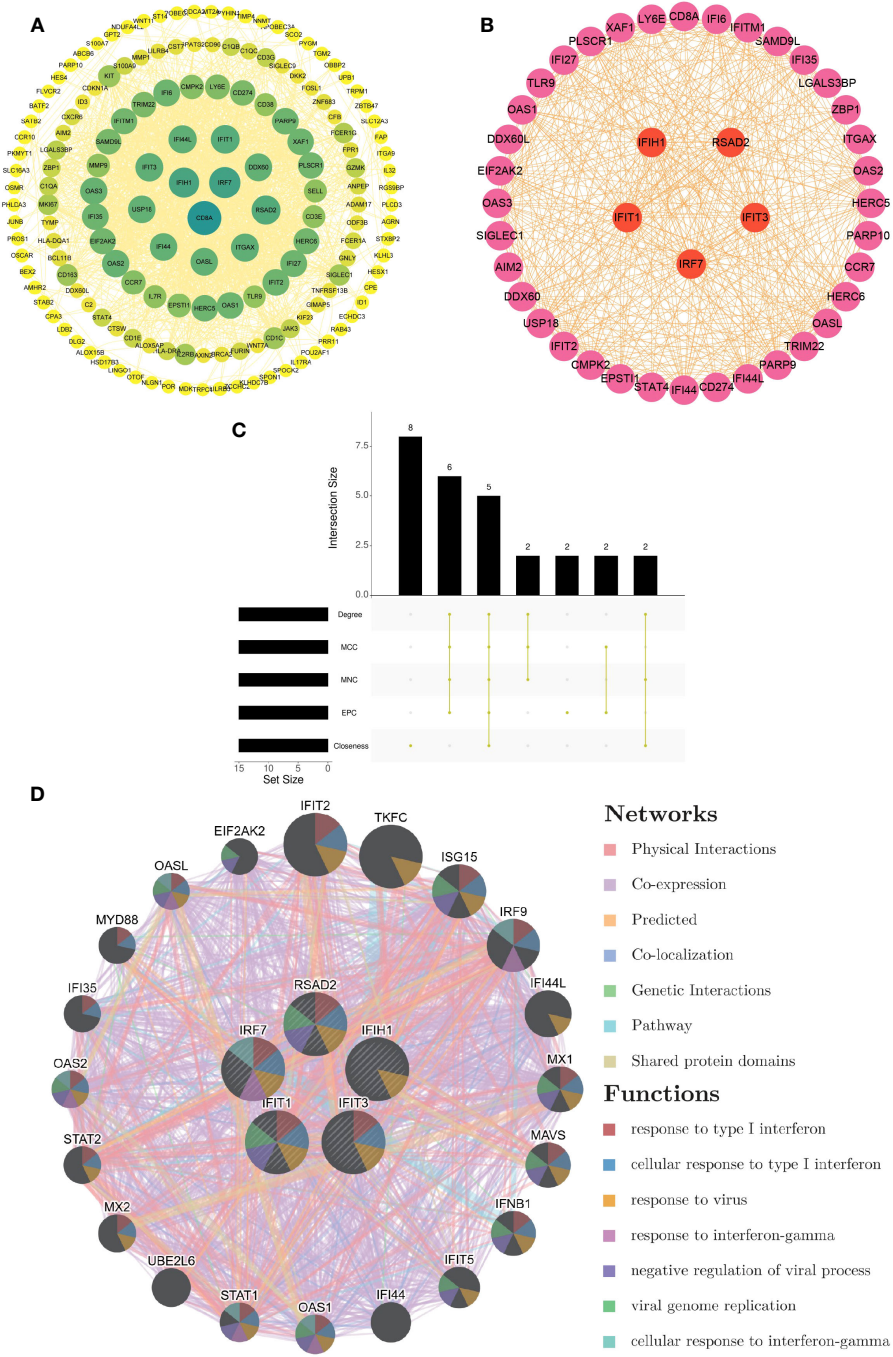
Development of PPI network and extraction of central genes **(A)** The visualization of the PPI network of shared DEGs was enabled by Cytoscape. The nodes correspond to related genes, and the lines denote the connectivity between nodes. **(B)** The network of the most crucial central genes. Each node signifies a distinct gene, and the lines depict the interplay among the nodes. **(C)** Extraction of central genes from the PPI network. The convergence of the top 15 genes from five disparate algorithms - Degree, MCC, MNC, EPC, and Closeness - uncovers the central genes. **(D)** Functional enrichment network of central genes. The colors of modules denote biological functions, while the colors of lines denote the connectivity between genes.

Based on the 214 common DEGs, we constructed a protein-protein interaction (PPI) network utilizing the STRING online database. Subsequently, we employed Cytoscape software to visualize the network, which revealed a total of 161 nodes and 1618 edges. The confidence score threshold was adjusted to 0.4 to ensure the reliability of the interactions (**Figure 3A**). To further identify the most influential genes within this network, we applied the cytoHubba plugin, which computed the top 15 genes based on their network centrality measures. Among these genes, a subsequent analysis using the UpSetR package allowed us to pinpoint five core genes of particular significance: IRF7, IFIH1, RSAD2, IFIT1, and IFIT3 (**Figures 3B, C**). These core genes play crucial roles in the network and are potential key regulators in the context of the analyzed conditions.

### Functional enrichment analysis of hub genes

From the GeneMANIA database, the functional interplay of the five pivotal genes (IRF7, IFIH1, RSAD2, IFIT1, and IFIT3) revealed diverse interaction forms. Physical interactions accounted for 77.64% of these interactions, co-expression relationships were 8.01%, predictions made up 5.37%, co-localization was 3.63%, genetic interactions were 2.87%, pathways contributed 1.88%, and shared protein domains made up 0.60%. In addition, the study underscored the critical functions of these core genes in numerous biological processes such as type I interferon responses, cellular activities in response to type I interferon, viral reactions, interferon-gamma responses, negative regulation of viral procedures, viral genome replication, and cellular reactions to interferon-gamma (**Figure 3D**).

### Construction of regulatory networks

**Figure 4:**
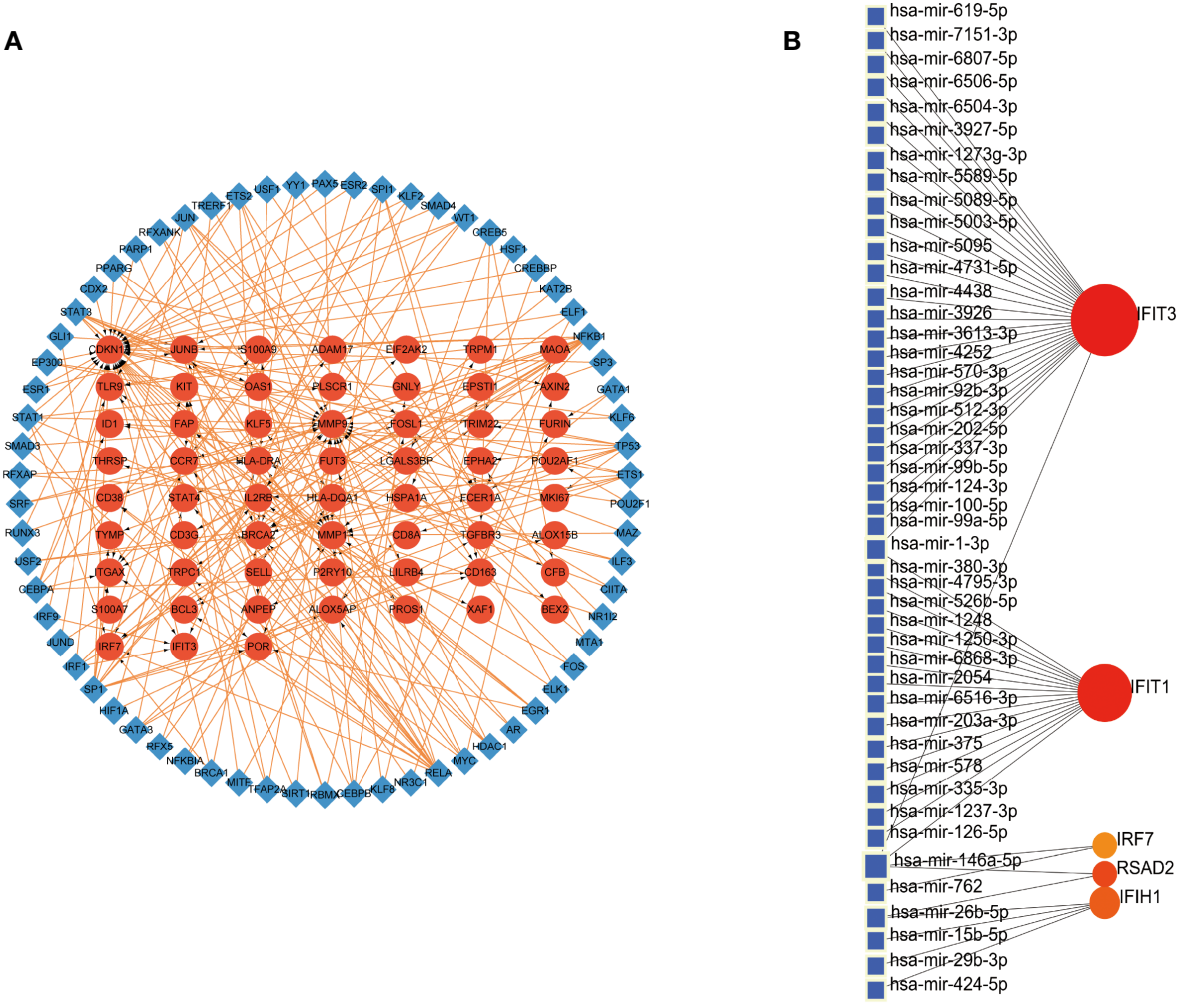
Construction of Regulatory Networks **(A)** Interaction network between TFs and DEGs. The network uses blue nodes to symbolize TFs, red nodes for DEGs, and directional arrows to indicate regulatory relationships. **(B)** The network of gene-miRNA interactions. The blue color nodes represent the miRNAs and the red color nodes represent hub genes.

We embarked on exploring the regulatory dynamics between TFs and common DEGs by building a TF-gene regulatory network using the TRRUST database. This network was brought to life with the help of Cytoscape software, featuring 66 TFs, 125 nodes, and 226 edges (See **Figure 4A**). To further examine the gene-miRNA regulatory network, we employed NetworkAnalyst. This led to a network model predicting the interactions between miRNAs and hub genes, comprising 5 hub genes, 51 nodes, and 51 edges (Refer to **Figure 4B**).

### Assessment of hub genes in diagnostic value

**Figure 5:**
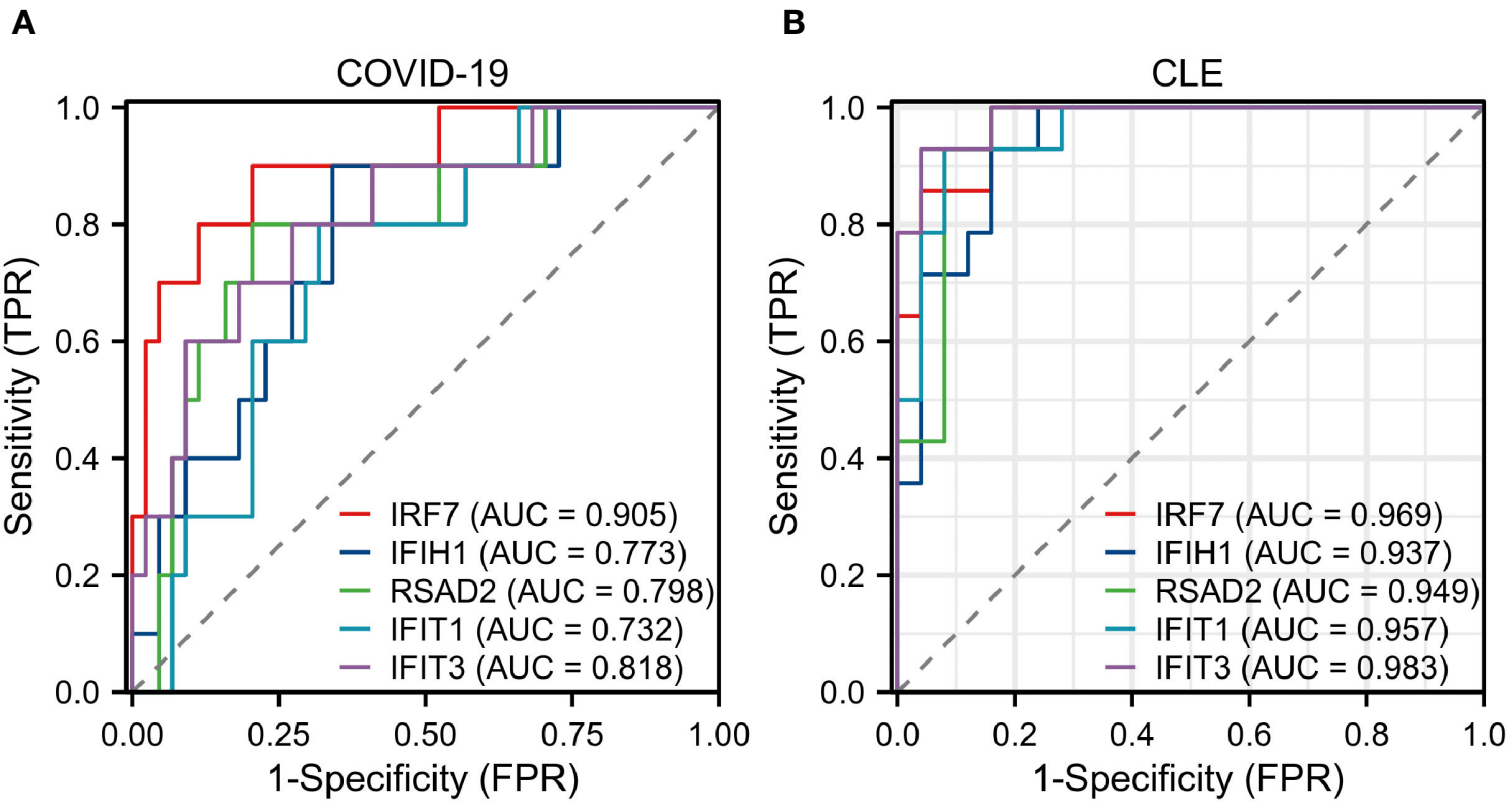
Assessment of hub genes in diagnostic value **(A)** The diagnostic efficacy verification in GSE171110. **(B)** The diagnostic efficacy verification in GSE109248.

The diagnostic validity of the five central genes was assessed by means of ROC curves. Both IRF7, with an AUC of 0.905, and IFIT3, having an AUC of 0.818, showcased commendable diagnostic proficiency in differentiating SARS-CoV-2 patients from individuals devoid of the disease (**Figure 5A**). Furthermore, IRF7 (AUC: 0.969), IFIH1 (AUC: 0.937), RSAD2 (AUC: 0.949), IFIT1 (AUC: 0.957), and IFIT3 (AUC: 0.983) put forth an impressive diagnostic performance when distinguishing CLE patients from the healthy control group (**Figure 5B**).

## Discussion

LE, an autoimmune disease that produces a range of symptoms across both CLE and SLE spectrums, demonstrates an intriguing parallelism with COVID-19 through overlapping immune responses (25). Among the underlying mechanisms breaking down immunological tolerance are molecular mimicry, bystander activation, and epitope spreading, all common to COVID-19 and ADs (26). Although SLE is not associated with the failure of COVID-19 vaccination and COVID-19 infection after vaccination, we found CLE/SLE-related clinical symptoms in people infected with COVID-19, so we tried to find possible research targets from two separate GSE171110 and GSE109248 data sets. An increasing number of case reports and research pieces suggest a possible heightened risk of LE development in association with COVID-19 infection and vaccination. Through a univariate analysis model study, Zecca et al. revealed that there was no correlation between SLE and vaccination failure (27)^;^ Rizzi et al. through univariate analysis showed that SLE was not associated with COVID-19 infection after the third dose of vaccine (28). Nevertheless, current broad-scale studies fall short of providing exhaustive clinical outcome data for COVID-19 patients simultaneously afflicted with CLE. Our research, therefore, intends to uncover the mutual molecular functions and pathways between COVID-19 and CLE, aiming to enhance the understanding of their interaction.

During this study, an exhaustive transcriptomic analysis was conducted, unearthing 214 shared DEGs between CLE and COVID-19. These shared DEGs underwent Gene Ontology enrichment analyses, which revealed significant enrichment in terms associated with defense responses to viruses, viral processes, double-stranded RNA binding, and immune receptor activity. The ongoing COVID-19 pandemic, driven by the positive single-stranded RNA virus SARS-CoV-2 (29), may utilize these biological processes discovered in our research as part of its infection strategy. During the replication of the virus, the formation of dsRNA replication intermediates can stimulate cytoplasmic innate immune pathways like MDA5 or RIG-I. This engagement initiates a signaling cascade through MAVS, leading to the production of type I and III interferons (IFNs). These IFNs, acting paracrinally and autocrinally, display antiviral functions both directly and indirectly (30). These findings are consistent with our study results, in which we identified enrichments in double-stranded RNA binding and immune receptor activity as molecular functions in COVID-19.

Our examination of the KEGG pathway analysis furnished 15 noteworthy enrichment pathways connected with the DEGs identified, notably Staphylococcus aureus infection, Coronavirus disease - COVID-19, Epstein-Barr virus infection, and others like Complement and coagulation cascades, along with Th1 and Th2 cell differentiation. Significantly, the pathway related to Coronavirus disease registered the maximum number of shared DEGs. Moreover, we discovered that these shared DEGs were prevalently enriched in a diverse set of infectious diseases including, but not limited to, staphylococcus aureus infection, Epstein-Barr virus infection, measles, and influenza. These results underline the possible role of both bacterial and viral infections in contributing to the origin of CLE and COVID-19.

Gene expression is modulated by a variety of elements at multiple levels, with critical roles being played by transcription factors (TFs) and microRNAs (miRNAs) in the control of transcription and post-transcriptional activities. It is acknowledged that the dynamic interplay between TFs and miRNAs, forming a comprehensive regulatory network, offers a potent approach to unravel the complex mechanisms of biological regulation (31). Our network analysis of TF-DEGs revealed the participation of several entities, namely IRF1, IRF9, SPI1, STAT1, NFKB1, RELA, KAT2B, BRCA1, and YY1, in the transcriptional regulation of hub genes IFIT3 and IRF7. Utilizing the TRRUST online tool, we performed a supplementary analysis and discovered that STAT1 emerges as a shared transcription factor for IFIT3 and IRF7. It is worth noting that studies have highlighted that IRF9, SPI1, and STAT1 partially contribute to the onset of CLE (31). With respect to the DEGs-miRNAs network analysis, we found several miRNAs implicated in the regulation of hub genes. Particularly noteworthy is research suggesting that miR-203 promotes epidermal differentiation by impeding proliferation potential and inducing cell cycle arrest in skin-related conditions (32). Recent investigations report an elevated expression of genes encoding IRF1, IRF5, IRF7, JAK2, and PML in severe COVID-19 patients. Interestingly, cells deficient in IRF1/STAT1, when stimulated by TNF-α and IFN-γ, demonstrated cell protective effects, mitigating cell death (33). Moreover, another study found increased expression of IRF1, STAT1, and IRF9 in moderate to severe COVID-19 patients in comparison with healthy subjects, suggesting these transcription factors are intimately involved in inflammatory damage tied to COVID-19 (34).

Upon constructing a protein-protein interaction network utilizing shared DEGs, five central genes emerged: IRF7, IFIH1, RSAD2, IFIT1, and IFIT3. The gene IRF7 (Interferon Regulatory Factor 7) is a significant transcriptional regulator essential for initiating the innate immune response against DNA and RNA viruses, and it plays an irreplaceable role in type I interferon (IFN)-dependent immune reactions (35). Examination of the genetic patterns in COVID-19 positive patients revealed a link between mutations in the IRF7 gene and COVID-19 mortality rates in African Americans, especially pronounced in elderly cohorts (36). In line with this, it was observed that individuals lacking IRF7 are prone to respiratory infections from COVID-19, yet otherwise maintain healthy states, hinting at a potential involvement of IRF7 in the development of COVID-19 (37). Another study showed that lupus’ keratinocytes exhibit a heightened response to interferon (IFN), emphasizing the crucial role of IRF7 in the progression of cutaneous lupus erythematosus (38). IFIH1, the gene that encodes for MDA5, acts as a cellular sensor for viral RNA, instigating the innate immune response (39). MDA5 was identified as the primary regulator of type I interferon (IFN) production during a SARS-CoV-2 infection (40). Moreover, it’s important to note the participation of IFIH1 in various inflammatory diseases. For instance, an enhanced IFIH1 expression is detected in the skin of chronic discoid lupus and lichen planus patients (41). RSAD2 encodes for an antiviral protein induced by interferon, contributing significantly to the cell’s antiviral state triggered by both type I and II interferons. It exerts inhibitory effects on a multitude of DNA and RNA viruses (42). Contemporary studies utilizing bioinformatics tools indicate a considerable upsurge in RSAD2 gene expression in COVID-19 or cutaneous lupus patients (43). This supports our analysis, suggesting RSAD2 as a potential shared therapeutic target for both conditions. The genes IFIT1 and IFIT3 encode for antiviral proteins stimulated by interferon. They have been shown to suppress various viral and cellular functions, such as cell proliferation, signaling, migration, and virus replication (44). An increased expression of IFIT1 and IFIT3 has been observed in SARS-CoV-2 infected cells, indicating an activation of the innate interferon response. Hence, these findings propose IFIT1 and IFIT3 as potential drug targets for the treatment of COVID-19 (45). Additionally, these molecules may potentially enhance the expression of CXCL10, a lymphocyte chemotactic factor, contributing to the inhibition of viral replication (46).

Past studies have separately examined key genes related to COVID-19 and CLE, but fewer have used bioinformatics to explore the shared molecular mechanisms between the two diseases. This study seeks to fill this gap by pioneering the identification and analysis of shared DEGs, hub genes, miRNAs, and TFs in both conditions. The data suggest that COVID-19 and CLE share pathogenic pathways potentially controlled by particular hub genes. And this research may pave the way for future studies aimed at understanding the complex molecular underpinnings of both COVID-19 and CLE pathologies.

## Data availability statement

The datasets presented in this study can be found in online repositories. The names of the repository/repositories and accession number(s) can be found below: https://www.ncbi.nlm.nih.gov/geo/under the accession numbers GSE109248/GSE171110.

## Ethics statement

GEO belongs to public databases. The patients involved in the database have obtained ethical approval. Users can download relevant data for free for research and publish relevant articles. Our study is based on open-source data, so there are no ethical issues or other conflicts of interest.

## Author contributions

Z-YG: Conceptualization, Data curation, Investigation, Software, Writing – original draft, Writing – review & editing, Methodology, Supervision. X-CZ: Methodology, Writing – review & editing. G-QY: Investigation, Methodology, Software, Validation, Writing – review & editing. YT: Methodology, Writing – review & editing. XH: Methodology, Writing – review & editing. Q-CW: Methodology, Software, Investigation, Writing – review & editing. L-CS: Conceptualization, Data curation, Formal Analysis, Investigation, Methodology, Project administration, Resources, Software, Supervision, Validation, Writing – review & editing. LY: Conceptualization, Supervision, Data curation, Investigation, Methodology, Software, Writing – review & editing.
